# Early Results with a New Posterior Chamber Phakic Intraocular Lens in Patients with High Myopia

**DOI:** 10.1155/2018/1329874

**Published:** 2018-06-19

**Authors:** Dilek Yaşa, Ufuk Ürdem, Alper Ağca, Yusuf Yildirim, Burçin Kepez Yildiz, Nilay Kandemir Beşek, Ulviye Yiğit, Ahmet Demirok

**Affiliations:** ^1^Beyoğlu Eye Research and Training Hospital, Bereketzade Mah., No. 2 Beyoglu, Istanbul, Turkey; ^2^Dr. Sadi Konuk Bakirkoy Research and Training Hospital, Zuhuratbaba Mah., Tevfik Saglam Cad., No. 21 Bakirkoy, Istanbul, Turkey

## Abstract

**Purpose:**

To report clinical results of a foldable, hydrophilic acrylic, single-piece, injectable, posterior chamber phakic intraocular lens (pIOL).

**Material and Methods:**

Medical records of patients who underwent posterior chamber phakic IOL (Eyecryl Phakic IOL, Biotech Vision Care, Ahmedabad, India) implantation for surgical correction of myopia were retrospectively reviewed. Only patients with at least a one-year follow-up were included. Manifest refraction, uncorrected distance visual acuity (UDVA), corrected distance visual acuity (CDVA), endothelial cell density (ECD), and pIOL vault were analyzed at 1, 3, 6, and 12 months after surgery. Complications observed during and after surgery were also recorded.

**Results:**

The study included 58 eyes of 29 patients. Mean patient age was 32 ± 7 years. Spherical equivalent of manifest refraction was −13.41 ± 3.23 D preoperatively and −0.44 ± 0.55 D postoperatively. Preoperative CDVA was 0.29 ± 0.71 logMAR. Postoperative UDVA and CDVA were 0.21 ± 0.66 and 0.15 ± 0.69 logMAR, respectively, at the 12-month visit. At the 12-month visit, the efficacy index was 1.20 and the safety index was 1.39. Mean ECD was 2713 ± 339 cells/mm^2^ at the preoperative visit and 2608 ± 362 cells/mm^2^ at the 12-month visit (3.9% loss, *p* < 0.001). ECD loss from 3 months to 12 months was not statistically significant. No significant cataract formation, significant endothelial cell loss, glaucoma, uveitis, or any other vision-threatening complication was observed.

**Conclusion:**

Based on postoperative experience, we have found that Eyecryl Phakic IOL is safe and effective for treating high myopia.

## 1. Introduction

Phakic intraocular lens (IOL) implantation, corneal refractive surgery (small incision lenticule extraction and laser in situ keratomileusis), and refractive lens exchange are alternatives for the surgical treatment of high myopia [1]. Refractive lens exchange may correct a high amount of myopia; however, it results in loss of accommodation (in addition to its potential complications); thus, it is not usually considered in nonpresbyopic individuals. Corneal refractive surgery is usually not considered for high myopia because the quality of vision decreases, and the complication rate increases after a certain degree. In addition, pIOL implantation may result a better quality of vision and offer significant vision-related quality-of-life advantages over corneal refractive surgery [2]. Phakic IOL implantation has the advantage of correcting greater myopia than corneal refractive surgery while preserving accommodation in contrast to refractive lens exchange.

A multitude of angle-supported and iris-fixated anterior chamber pIOLs have been taken off the market because of excessive endothelial cell loss and complications such as cataract, glaucoma, and pupil ovalization [3, 4]. However, some models of posterior chamber pIOL and iris-fixated pIOL are considered to have good safety and efficacy, yielding predictable and stable results [5–10]. Today, there are only a few commercially available options for posterior chamber and iris-supported pIOLs on the market. Eyecryl Phakic IOL (Biotech Vision Care, Ahmedabad, India) is a foldable, hydrophilic acrylic, single-piece, injectable, posterior chamber phakic IOL and, to the best of our knowledge, there are no published studies describing clinical outcomes following implantation of this lens.

In this study, we evaluated the efficacy and safety of Eyecryl posterior chamber phakic IOL implantation in patients with high myopia.

## 2. Patients and Methods

This study followed the tenets of the Declaration of Helsinki, and approval was obtained from the Ethics Committee of Bakirkoy Research and Training Hospital. At the time of the surgery, all patients were fully informed about the details and possible risks of the surgical procedure. Written informed consent was obtained from all patients before surgery. Medical records of patients who underwent Eyecryl Phakic IOL implantation were retrospectively evaluated. Only the patients with at least a 1-year follow-up were included in the study. The main outcome measures in this study were the spherical equivalent (SE) of manifest refractive error, UDVA, CDVA, and ECD at 1 month, 3 months, 6 months, and 1 year after surgery. Perioperative and postoperative complications were also recorded, giving special attention to cataract development. We defined cataract as a lens opacity of any type that results in the loss of ≥2 lines of CDVA or cataract surgery.

### 2.1. Preoperative and Postoperative Examinations

All patients underwent the standard detailed anterior and posterior segment examination procedure of our Refractive Surgery Clinic preoperatively and postoperatively. All patients were examined at postoperative day 1; week 1; and months 1, 6, and 12 because it is routine in our clinic. The patients were scheduled for yearly follow-up thereafter.

An autorefractometer (RM-8800 Autorefractor, Topcon, Tokyo, Japan) was used for keratometry measurements and objective refraction. An automated phoropter (CV-5000, Topcon, Tokyo, Japan) and a back-illuminated 19″ LED LCD monitor chart (CC-100 XP, Topcon, Tokyo, Japan) were used for uncorrected distance visual acuity (UDVA) and corrected distance visual acuity (CDVA) measurements. Visual acuities were converted to logMAR for statistical analysis. Corneal topography and corneal pachymetry mapping were performed with the Sirius topography platform (Schwind eye-tech-solutions GmbH, Germany). Endothelial cell density was measured with a specular microscope (CEM 530, NIDEK, Japan). Intraocular pressure was measured with a Goldmann applanation tonometer at every visit. All patients underwent a detailed anterior and posterior segment examination with a slit lamp. All these examinations were performed in preoperative and all postoperative visits except for the first postoperative day, when only UDVA, CDVA, slit lamp, and IOP measurements were performed. In addition, in all postoperative visits, the phakic IOL vault (distance between the phakic lens and the crystalline lens) was measured with an anterior segment optical coherence tomography (OCT) device (Visante OCT, Carl Zeiss AG, Germany). In our clinic, it is routine to implant pIOLs only in patients with an anterior chamber depth of at least 3 mm from the endothelium. Anterior chamber depth from the endothelium and white-to-white measurements was measured with an IOL Master (Carl Zeiss Meditec, Germany).

### 2.2. Phakic Intraocular Lens and Surgical Procedure

The Eyecryl Phakic IOL is a foldable, hydrophilic acrylic, single-piece, injectable, posterior chamber phakic IOL. It is designed to be placed in the posterior chamber behind the iris with the haptic zone resting on the ciliary sulcus. It is available in 3 overall lengths (12.0 mm, 12.5 mm, and 13.0 mm) and is designed to correct myopia in a dioptric power range of −3.00 to −23.00 diopters (D). It has an aspheric optic with zero aberration. The diameter of the optic is 4.65 to 5.50 mm. A 320 *µ*m hole in the center of the optic prevents pupillary block and improves aqueous humor circulation. Power calculation for the phakic intraocular lens was performed using the modified vergence formula in the software provided by the manufacturer. Target was emmetropia in all cases. The lens size was determined based on the horizontal white-to-white (WTW) distance.

All surgeries were performed by the same surgeon (AA). The pupil was dilated with cyclopentolate and phenylephrine drops, instilled 30 minutes prior to surgery. After sub-Tenon anesthesia, a 2.8 mm clear corneal tunnel incision was performed in the horizontal temporal meridian. The anterior chamber was filled with sodium hyaluronate 1%. The Eyecryl Phakic IOL was implanted behind the iris through the incision, using the injector cartridge supplied by the manufacturer. A temporal clear corneal incision was used in all cases. As a result, the position of the pIOLs was horizontal immediately after implantation. To avoid any unnecessary trauma to intraocular structures (i.e., the crystalline lens, iris, ciliary sulcus, and zonula), pIOLs were left in this horizontal position. After the Eyecryl Phakic IOL was gently positioned in the sulcus, the remaining viscoelastic material was completely washed out of the anterior chamber with a balanced salt solution, and a miotic agent was instilled. No preoperative or intraoperative peripheral iridectomies were performed.

### 2.3. Statistical Methods

Statistical analysis and the associated tables and listings were performed using SAS®, version 9.4. Descriptive statistics were obtained. The assumption of normality was assessed by the Shapiro–Wilk test. If *p* value was >0.05, it was assumed that the data followed a normal distribution. Paired *t*-test was used to analyze data with normal distribution, and nonparametric Wilcoxon signed rank test was used to analyze the data with a non-normal distribution. One-way analysis of variance (ANOVA) was used to evaluate ECD and the vault changes over time.

## 3. Results

**Figure 1:**
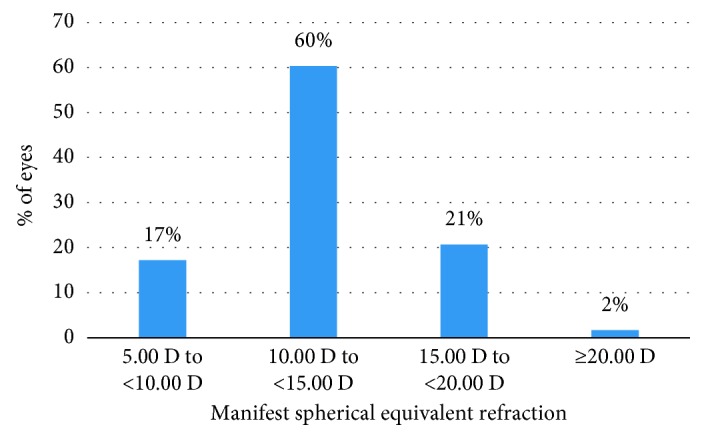
Distribution of manifest spherical equivalent for the patients preoperatively.

Fifty-eight eyes of 29 subjects were included in the study. Among 29 subjects, 6 (21%) subjects were male and 23 (79%) subjects were female. All patients had pIOL implantation bilaterally. Preoperative characteristics and distribution of preoperative SE of manifest refraction are shown in Table 1 and Figure 1, respectively.

**Figure 2:**
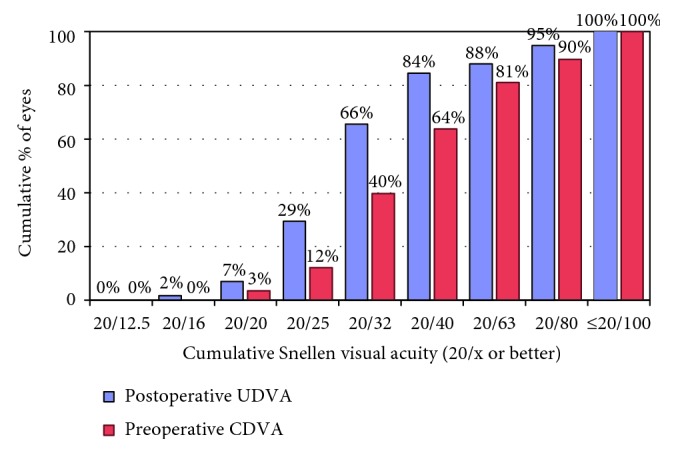
Postoperative uncorrected distance visual acuity (UDVA) versus preoperative corrected distance visual acuity (CDVA).

**Figure 3:**
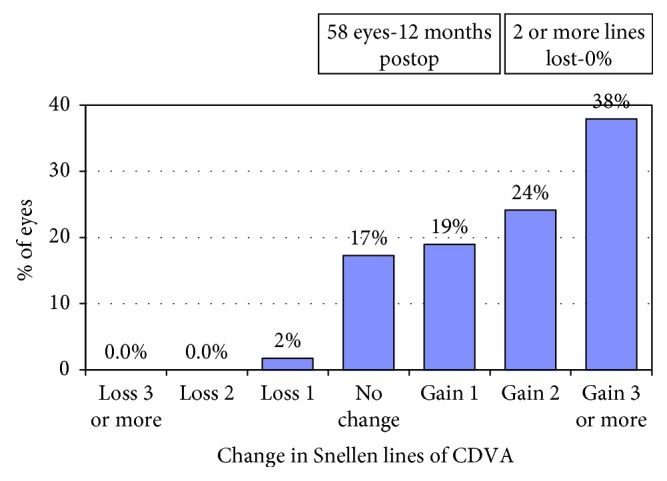
Change in corrected distance visual acuity (CDVA).

Mean preoperative CDVA was 0.29 ± 0.69 logMAR. Mean UDVA was 0.20 ± 0.66 logMAR at 1 month, 0.21 ± 0.65 logMAR at 3 months, 0.18 ± 0.68 logMAR at 6 months, and 0.21 ± 0.66 logMAR at 12 months. Mean UDVA at 1 month was statistically significantly better than mean preoperative CDVA (paired samples *t*-test, two-tailed, *p* < 0.001). Efficacy index (ratio of postoperative CDVA to preoperative UDVA) was 1.20 at 1 year.  Figure 2 shows preoperative and postoperative cumulative Snellen visual acuity (preoperative CDVA and postoperative UDVA). Preoperative CDVA was 0.29 ± 0.69 logMAR, and postoperative CDVA was 0.15 ± 0.69 at the last follow-up visit (1 year, paired sample *t*-test, two-tailed, *p* value <0.001). The safety index (ratio of postoperative CDVA to preoperative CDVA) was 1.39 at 1 year. No patient lost 2 or more lines of CDVA, and 62% of the eyes gained 2 or more lines of CDVA (Figure 3).

**Figure 4:**
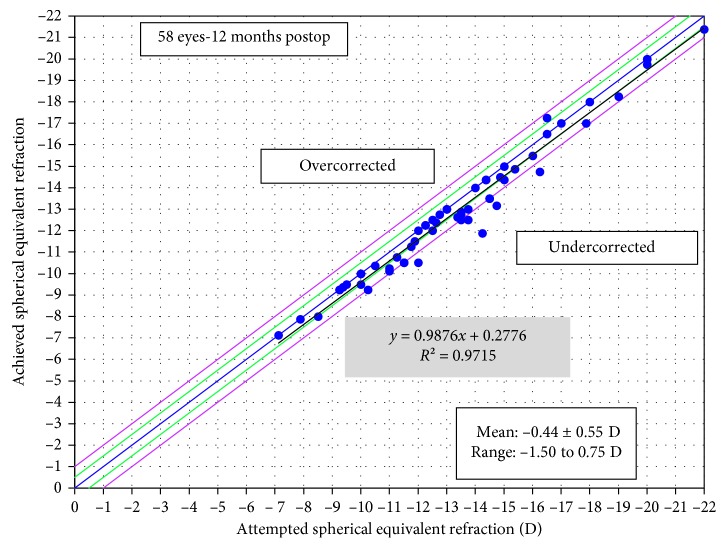
Attempted versus achieved spherical equivalent of manifest refraction.

**Figure 5:**
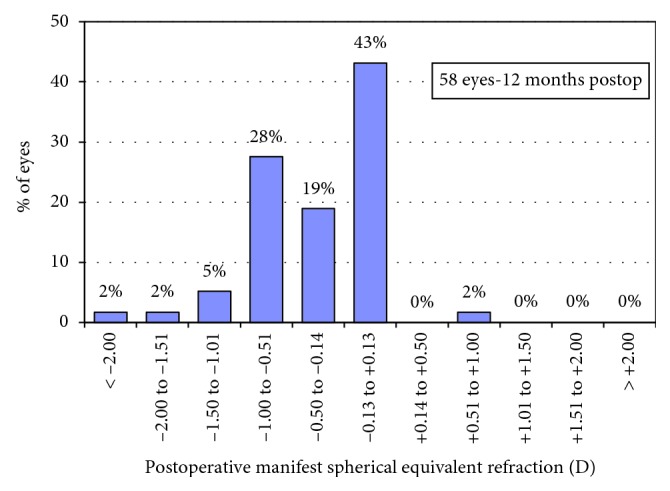
Distribution of manifest spherical equivalent for the patients postoperatively.

**Figure 6:**
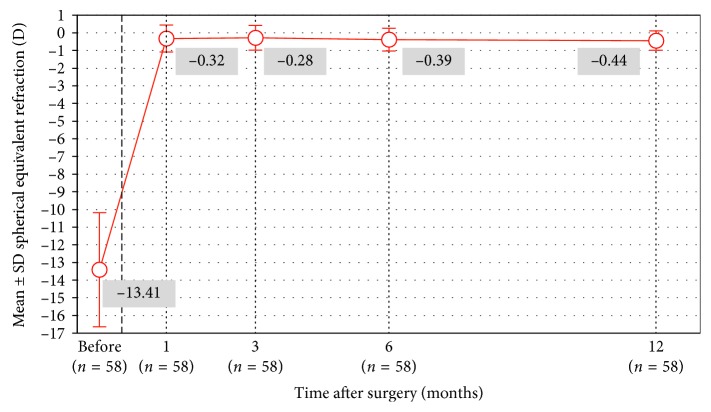
Mean manifest refraction throughout follow-up.

Figure 4 shows the attempted versus achieved refractive correction. At 12 months, 62% of the eyes were within ±0.50 D of the attempted correction, and 93% of the eyes were within ±1.00 D of the attempted correction (Figure 5).  Figure 6 shows the stability of manifest refraction throughout follow-up.

**Figure 7:**
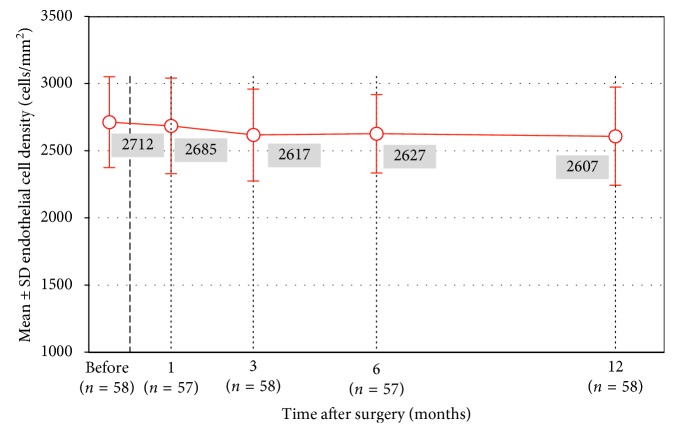
Mean endothelial cell count (*n*=58, repeated measures ANOVA, all visits, *p* < 0.001). Statistically significant difference was observed from preoperative visit to postoperative month 3 (*p*=0.001), postoperative month 6 (*p* < 0.001), and postoperative month 12 (*p*=0.003). No statistically significant difference was observed from preoperative visit to postoperative month 1 (*p*=0.1267). ECD loss from three months to 12 months was not statistically significant (*p*=0.674).

Figure 7 shows the mean ECD at preoperative and different postoperative visits. ECD was 2713 ± 339 cells/mm^2^ at the preoperative visit, and 2608 ± 362 cells/mm^2^ at the 12-month visit (3.9% loss, paired sample *t*-test, *p* < 0.001). ECD loss from 3 months to 12 months was not statistically significant.

**Figure 8:**
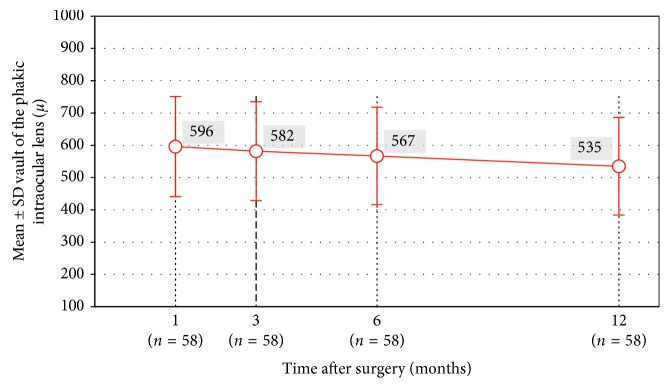
Mean vault of the phakic IOL (*n*=58, repeated measures ANOVA, all visits, *p* < 0.001). Statistically significant difference was observed from the 1-month visit to the 6-month visit (*p*=0.04) and from the 6-month visit to the 12-month visit (*p* < 0.001).

Figure 8 shows the mean vault of the pIOL during follow-up. The mean vault was 535 ± 137 (min: 270; max: 880) at 1 year. There was a statistically significant decrease in vault during follow-up (repeated measures ANOVA, *p* < 0.001). At the 12-month visit, the vault of the pIOL had decreased 57 ± 91 *µ* when compared to the 1-month visit (paired sample *t*-test, *p* < 0.001).

The mean preoperative central corneal thickness (thinnest) was 530 ± 33.26 *µ*m. Postoperatively, the mean central corneal thickness (thinnest) was 532 ± 30.86 *µ*m at 1 month, 529 ± 32.95 *µ*m at 3 months, 528 ± 31.83 *µ*m at 6 months, and 530 ± 32.18 *µ*m at 12 months. No statistically significant difference was observed from preoperative visit to all postoperative visits (repeated measures ANOVA, *p*=0.9703).

### 3.1. Complications

In both eyes of one patient, elevated intraocular pressure (IOP) (24 mmHg bilaterally) was detected at the one-month visit. The increase in IOP was considered steroid induced because there was no pupillary, block, inflammatory reaction, or pigment dispersion. The intraocular pressures returned to their baseline levels after the cessation of topical steroid treatment. There were no cases of anterior subcapsular cataracts or opacities. There were no other intraoperative or postoperative complications.

## 4. Discussion

There are no published studies on the results of the IOL implanted in this study, and the amount of myopia in this study is higher when compared to other posterior chamber phakic IOL studies in the literature [2, 6–16]. In a multicenter, prospective study on refractive surgery in 15,011 eyes reported by Kamiya et al. [6], a pIOL with a very similar design (plate haptic posterior chamber IOL with a central hole) was implanted in 1319 eyes. They reported that the mean patient age was 32 years, and the mean SE was −8.42 ± 3.10 D. In our study, the mean patient age was similar (31 years), but the mean SE of manifest refraction was −13.41 ± 3.22 D, and 23% of our patients had myopia higher than −15.00 D. The refractive error in our study was relatively high because this retrospective case series reflects the practice in our clinic. We prefer implantation of phakic IOLs only in nonpresbyopic patients who are not suitable for corneal refractive surgery (mainly small incision lenticule extraction).

As expected, we found that SE decreased and UDVA increased after implantation of phakic IOL. We obtained predictable postoperative refractive results in line with previous studies on other types of phakic IOLs. At 12 months, 62% of the eyes were within ±0.50 D of the attempted correction, and 93% of the eyes were within ±1.00 D of the attempted correction. Lee et al. [8] reported that in a series of 281 eyes, 69% and 87.2% were within ±0.50 D and ±1.00 D of the desired refraction 5 years after surgery, respectively. Alfonso et al. [12] reported that 86.7% were within ±0.50 D one month after surgery. However, only 64.1% and 38% were within ±0.50 D 3 and 5 years after surgery, respectively. Our patient groups had a considerably higher level of mean SE and lower DCVA than in other studies in the literature. For example, Huseynova et al. [14] reported that 75% and 100% were within ±0.50 D and ±1.00 D of the desired refraction 3 months after surgery, respectively. However, the CDVA of their patients was 0.12 logMAR (decimal notation: 0.76), whereas the mean preoperative CDVA in our patients was 0.29 logMAR (decimal notation: 0.51). We believe that the percentage of eyes within ±0.50 D would be higher if SE were lower and DCVA were better in our patient group. In contrast to cataract surgery, refractive vergence formulas are used to determine the power of the IOL to be implanted in phakic eyes. As a result, precise determination of manifest refraction is critical in these patients to calculate the pIOL to correct that manifest refractive error [17]. However, precise determination of manifest refraction gets more difficult as the CDVA decreases. For example, a patient with a visual acuity of 20/40 may not respond to 0.50 D changes during subjective manifest refraction.

In this study, we found the efficacy index to be 1.20, indicating that the mean postoperative UCVA was better than preoperative CDVA after implantation of Eyecryl Phakic IOL. Lisa et al. [10] evaluated a posterior chamber pIOL with a similar design and a central hole implanted in 147 eyes and found an efficacy index of 1.04, whereas Shimizu et al. [13] and Cao et al. [7] found efficacy indices of 1.13 and 1.11, respectively, with the same lens model. In addition to the expected increase in UDVA, there was an improvement in CDVA of the patients. Eighty-one percent of the eyes gained 1 line, 62% percent of the eyes gained 2 or more lines of CDVA, and none of the eyes lost 2 or more lines; the safety index was 1.39. Previous studies have also found that 35% to 100% of the pIOL implanted in patients experienced 1 or more lines of CDVA increase after pIOL implantation [5–10, 12–14, 18, 19]. Although the exact mechanism is unclear, the relative magnification of the image and the reduction in visual aberrations after an anterior chamber pIOL implantation when compared to spectacle lenses may be the reason for the improvement [20]. In addition, the studies in the literature that evaluate CDVA improvement in adult amblyopic eyes after laser-assisted in situ keratomileusis (LASIK), photorefractive keratectomy (PRK), or pIOL implantation agree that improvement occurs; however, the rates vary over a wide range [19, 21, 22].

The amount of endothelial cell loss after pIOL implantation has always been a topic of discussion. In the present study, the cumulative mean percentage of endothelial cell loss was 3.9% at 3, 6, and 12 months after the surgery. The most detailed data with the highest level of evidence on the ECD loss after a posterior chamber pIOL (Visian implantable Collamer lens, STAAR Surgical, Nidau, Switzerland) were reported during the prospective, multicenter U.S. FDA trial and showed that it was 3.3 ± 7.6% at one year (90% confidence limits: 2.4% to 4.3%) and 9.7 ± 9.3 at 4 years [11, 23]. Moya et al. [16] published a cumulative 12-year retrospective study, including data from 144 eyes implanted with the same pIOL model of implantable contact lenses (ICLs) between 1998 and 2001 and estimated a 6.46% surgically induced ECD decrease during the first year and an average yearly decrease rate of 1.20% after that. Other studies in the literature report much lower levels of ECD loss during the first year; they all agree that endothelial damage occurs primarily during the surgical procedure, and the rate of ECD loss decreases after a certain time [10, 12, 15, 24]. In line with all these studies, absence of a statistically significant difference in ECD after three months postoperatively in this study reflects the fact that the power of the statistical test is insufficient to reveal the small amount of normal endothelial loss. On the contrary, detection of an acute loss early after the surgery and stabilization after the 3-month visit suggests that the main reason for the cell loss is surgical trauma and that the pIOL does not induce a *clinically* significant amount of ECD loss by itself, *at least in the patients included in this study*. Previous studies show that a smaller ACD is a significant risk factor for increased ECD loss in both anterior and posterior chamber pIOLs [11, 25]. Accordingly, it is routine in our clinic to implant pIOLs of any type in only patients who have a ACD greater than 3.00 mm and the minimum anterior chamber depth (ACD) in this study was 3.04 mm from the endothelium.

Cataract formation is another major concern when implanting a pIOL in a young, highly myopic patient because cataract surgery results in loss of accommodation and increases the rate of retinal complications. Asymptomatic anterior subcapsular lens opacities (ASCLO) are recorded between 0% and 18% after surgery, and the difference is probably related to surgical technique, the retrospective nature of the studies, and to the definition of cataract. In this retrospective study, we defined cataract as a lens opacity of any type that results in loss of ≥2 lines of CDVA or cataract surgery. None of the patients in this study had a cataract at the 1-year visit. Also, there were no cases of anterior subcapsular opacities. However, because of the retrospective nature of the study, very mild anterior subcapsular opacities without clinical significance could have gone unnoticed and could only have been revealed in a prospective study. This result is in line with previous reports that the incidence of a visually significant cataract after pIOL implantation is low in the early postoperative years [26]. However, recent studies show that the rate of cataract formation is higher in longer follow-up. In a retrospective study, Lee et al. [8] reported that 2.1% of 281 eyes in their study developed a cataract at 5 years. Guber et al. [27] reported that phacoemulsification was performed in 4.9% and 18.3% of 133 eyes at 5 and 10 years after ICL implantation, respectively. Low vault, higher levels of myopia, and older patient age are risk factors for cataract formation after pIOL implantation [28].

Extremes of vault are risk factors for complications such as cataract, pigment dispersion, pupillary block, and glaucoma. However, precise definitions of excessive and insufficient vault are not clear. In the literature, the lower limit of safe vault is reported to be between 50 and 250 *µ* by different authors, and the upper limit is around 1000 *µ*, as long as the anterior chamber structure and pupillary function are normal [10, 28–30]. However, given the yearly increase in crystalline lens rise and the young age of the patients, we believe that it is advisable to be as close to 250 *µ* as possible. The lens vault is closely related to appropriate sizing of the pIOL to the posterior chamber. Mean vault in our patient group was 535 ± 137 (min: 270; max: 880) at 1 year, indicating that the sizes of the pIOLs matched well with the posterior chambers of the patients in our study.

In line with the previous studies on posterior chamber phakic IOLs, there were no sight-threatening intraoperative or postoperative complications in our patients up to 1 year after surgery.

The only complication was a bilateral, steroid-induced, transient IOP rise in one patient. Glaucoma may occur after anterior or posterior pIOL implantation due to pupillary block or pigment dispersion. Although preoperative or intraoperative peripheral iridectomies were not performed in our patients, no pupillary block was seen during follow-up. This is probably due to the central hole in the optic, which prevents pupillary block despite the lack of a peripheral iridectomy. In addition, no pigment dispersion or pigment dispersion glaucoma was observed. However, gonioscopy was not performed preoperatively and postoperatively because this study was retrospective, and gonioscopy was not a routine part of our preoperative and postoperative examinations. Thus, very mild clinical pigment dispersion in some patients could have gone unnoticed.

The weak points of this study are its retrospective nature and the relatively short follow-up to draw conclusions on two specific issues: long-term endothelial safety and rate of cataract formation at long term. However, it would take many years before the exact incidences of these two complications are revealed, and we would like to underline the fact that no conclusions can be drawn regarding long-term ASC formation based on the short 12-month follow-up used in the study.

We have found that Eyecryl Phakic IOL is safe and effective for treating high myopia, similar to other models of posterior chamber IOLs. Prospective studies with larger patient groups and longer follow-up are needed to reveal long-term efficacy and safety.

## Figures and Tables

**Table 1:** Preoperative patient characteristics.

	Mean ± SD	Minimum	Maximum
Age (years)	31 ± 6.92	23	49
SE (D)	−13.41 ± 3.22	−7.13	−22.00
Cylinder (D)	1.10 ± 0.70	0	2.25
DCVA (logMAR)	0.29 ± 0.72	1	0
WTW (mm)	11.72 ± 0.30	10.82	12.10
ECD (cells/mm^2^)	2712 ± 338.50	2048	3227
ACD (mm)	3.63 ± 0.21	3.04	4.04
Mean Sim K (D)	44.48 ± 1.72	39.21	47.49
IOP (mmHg)	14 ± 2.35	10.00	21.00
AL (mm)	28.23 ± 1.23	24.15	31.12
Corneal thickness (*µ*)	530 ± 33.27	452	595

SE: spherical equivalent; DCVA: corrected distance visual acuity; WTW: white-to-white; ECD: endothelial cell density; Sim K: simulated keratometry; IOP: intraocular pressure; AL: axial length.

## Data Availability

The datasets used and/or analyzed during the current study are available from the corresponding author on reasonable request.
